# Ximelagatran *versus* warfarin for prophylaxis of venous thromboembolism in major orthopedic surgery: systematic review of randomized controlled trials

**DOI:** 10.1590/S1516-31802006000600012

**Published:** 2006-11-01

**Authors:** Winston Bonetti Yoshida, Regina Paolucci El Dib, Ricardo de Alvarenga Yoshida, Francisco Humberto de Abreu Maffei

**Keywords:** Venous thrombosis, Pulmonary embolism, Orthopedics, Primary prevention, Meta-analysis, Trombose venosa, Embolia pulmonar, Ortopedia, Prevenção primária, Metanálise

## Abstract

**BACKGROUND:**

Ximelagatran has been recently studied for prophylaxis in surgical orthopedic cases.

**PURPOSE:**

We proposed to establish whether interventions involving ximelagatran, as compared with warfarin, would increase thromboembolic prophylaxis in patients undergoing major orthopedic knee surgery.

**DATA SOURCE:**

Studies with random assignment were identified by an electronic search of the medical literature up to 2006. Data were doubleentered into the Review Manager software, version 4.2.5.

**DATA SYNTHESIS:**

We included three wellconducted clinical trials involving 4,914 participants. Sub-groups with two dosages of ximelagatran (24 mg and 36 mg, b.i.d.), were defined. Ximelagatran showed significantly lower frequency of total venous thromboembolism (VTE) than warfarin, but only with the 36-mg dosage (risk relative, RR: 0.72; 95% confidence interval, CI: 0.64-0.81; p < 0.00001). For the 24-mg subgroup, total VTE frequency was similar (RR: 0.86; 95% CI: 0.73-1.01; p = 0.06). No significant differences were shown with either ximelagatran dosage for deep vein thrombosis (DVT), pulmonary embolism, any bleeding or severe bleeding. At the end of the treatment, alanine aminotransferase (ALT) elevation was less frequent in the 24-mg ximelagatran sub-group (RR: 0.33; 95% CI: 0.12-0.91; p = 0.03], but during the follow-up period, the ALT elevation rate was greater in the 36-mg ximelagatran group (RR: 6.97; 95% CI: 1.26-38.50; p = 0.03].

**CONCLUSIONS:**

Ximelagatran appears to be more effective than warfarin when used in higher dosages (36 mg b.i.d.), but at the expense of increased frequency of ALT elevation during the follow-up period.

## RESUMO

**INTRODUÇÃO:**

O ximelagatrano foi recentemente estudada para profilaxia do tromboembolismo venoso (TEV).

**OBJETIVO:**

Avaliar se o ximelagatrano comparado com a varfarina melhora a profilaxia do TEV em pacientes submetidos à cirurgia ortopédica do joelho.

**FONTE DE DADOS:**

Estudos randomizados identificados por pesquisa eletrônica na literatura médica, até 2006, cujos dados foram compilados no programa Review Manager, versão 4.2.5.

**RESULTADOS:**

Foram incluídos três estudos randomizados bem conduzidos envolvendo 4.914 participantes. Foram definidos dois sub-grupos com dosagens diferentes de ximelagatrano (24 mg and 36 mg, duas vezes ao dia). O tratamento com ximelagatrano mostrou freqüência significantemente menor de TEV que o tratamento com varfarina, mas somente na dosagem de 36-mg [risco relativo, RR 0.72 ([intervalo de confiança, IC, 95% 0.64, 0.81), p < 0.00001]. A freqüência de TEV no sub-grupo de 24-mg foi similar a da varfarina [RR 0.86 (IC 95% 0.73, 1.01), p = 0.06]. Para TEV maior, embolia pulmonar, sangramento e sangramento maior não houve diferença entre varfarina e a ximelagatrano. Ao final do tratamento, a elevação da alanino-aminotransferase (ALT) foi menos freqüente no sub-grupo de 24 mg de ximelagatrano que no grupo da varfarina [RR 0.33 (IC 95% 0.12, 0.91) p = 0.03], mas no período de acompanhamento essa elevação foi maior com 36 mg de ximelagatrano [RR 6.97 (IC 95% 1.26, 38.50) p = 0.03].

**CONCLUSÃO:**

O ximelagatrano foi mais efetivo que a varfarina quando usado em dosagens maiores (36 mg, 2 vezes ao dia), mas às expensas de aumento de enzimas hepáticas no período de acompanhamento.

## INTRODUCTION

Venous thromboembolism (VTE) is a common complication among hospital inpatients. Those undergoing major orthopedic surgery, which includes hip and knee arthroplasty and hip fracture repair, are a group that is at particularly high risk of VTE, and routine thromboprophylaxis is mandatory. The rates of venographic deep vein thrombosis (DVT) and proximal DVT, seven to fourteen days after major orthopedic surgery in patients receiving no prophylaxis, are approximately 40 to 60% and 10 to 30%, respectively.^1,2^

Guidelines recommend the use of anticoagulants for the prevention of VTE in these cases, but this treatment has some limitations. Oral vitamin K antagonists (VKA) are subject to several drug interactions and need careful monitoring. Low-molecular weight heparins (LMWH) are apparently more beneficial than unfractionated heparin (UH) and warfarin, but they require subcutaneous administration. This is inconvenient for some patients and may be associated with higher risk of bleeding than is warfarin, especially if heparin therapy is started soon after surgery.^2^

More recently, a new generation of anticoagulants has been tested for major orthopedic surgery prophylaxis. Randomized clinical trials have shown that recombinant hirudin beginning just before surgery is more efficacious than low-dose unfractionated heparin (LDUH)^3,4^ and LMWH,^5^ with no differences in bleeding. However, hirudin has not been approved for prophylactic use in the United States. Fondaparinux, a synthetic subcutaneously injected pentasaccharide similar to LMWH, has been found to be more effective than VKA in preventing asymptomatic and symptomatic in-hospital VTE by indirect comparison, and has been approved for prophylaxis in the United States.^2^ The most recent anticoagulant presented is ximelagatran, an oral direct antithrombin inhibitor. It is rapidly absorbed and converted to its active form, melagatran, which can also be applied subcutaneously.^6^ It has been studied mainly in orthopedic surgery prophylaxis in comparison with LMWH or warfarin.^7^

To this date, there have been two systematic reviews summarizing the results of these trials. Both of these meta-analyses compared sub-groups dealing with the timing of initial administration of melagatran/ ximelagatran *versus* LMWH.^8,9^ We therefore proposed to establish whether interventions involving ximelagatran would increase thromboembolic prophylaxis in patients undergoing major orthopedic knee surgery, in comparison with warfarin.

## METHODS

### Literature search

We searched the Cochrane Peripheral Vascular Diseases group records (up to May 2005), the Cochrane Central Register of Controlled Trials (Central, The Cochrane Library, issue 2, 2005), Medical Literature Analysis and Retrieval System Online (MEDLINE; 1966 to May 2005), Excerpta Medica database (EMBASE; 1980 to May 2005) and Literatura Latino-Americana e do Caribe em Ciências da Saúde (LILACS; 1982 to May 2005), to identify randomized controlled trials.

The databases were searched using a comprehensive search strategy for randomized controlled trials, along with MeSH (medical subject headings) and text words, including the following exhaustive list of synonyms: melagatran, ximelagatran, prophylaxis, thromboembolism, venous thrombosis, thrombophlebitis and pulmonary embolism. Unpublished studies were sought by contacting the AstraZeneca company and conducting structured internet searches. The bibliographic references of relevant review articles were also examined for eligible trials. References to the relevant studies identified and advertisement folders were also scrutinized for additional citations.

### Data Collection

Two reviewers (WBY and RAY) independently screened the trials identified through the literature search, extracted the data, assessed trial quality and analyzed the results. Two other reviewers (RPED and FHAM) were consulted whenever there was any disagreement. If consensus was not reached, data from the trials in question were not included unless or until the authors of the trial were able to resolve the contentious issues.

A standard form was initially used to extract the following information: characteristics of the study (design and randomization methods); participants; interventions; and outcomes (types of outcome measurements, timing of outcomes and adverse events).

### Endpoints

The primary outcome measurement was the frequency of total VTE: DVT at any site; pulmonary embolism (PE); or unexplained death during treatment. The secondary outcome measurements were:

- Frequency of major VTE (proximal DVT, PE or unexplained death);
- Frequency of any bleeding;
- Frequency of severe bleeding: number of patients with severe bleeding, i.e. bleeding involving a critical site (intraocular, intraspinal, pericardial, or retroperitoneal), excessive bleeding as judged by the investigator, or other bleeding not meeting these criteria, but classified as severe in the central adjudication;
- Volume of blood loss during surgery (mean ± standard deviation, SD);
- Volume of whole blood or red blood cells transfused (mean ± SD);
- Frequency of patients receiving transfusions;
- Bleeding index: number of units of red blood cells transfused plus the difference between pre-bleeding hemoglobin level in g/dl and post-bleeding event hemoglobin level in g/dl);
- Frequency of alanine aminotransferase (ALT) elevation to three times above the upper normal limit;
- Death (due to any cause) during the study period and follow-up (up to six months after surgery);
- Follow-up.

### Assessing methodological quality

The methodological quality of the trials included in this review was judged using the Cochrane instrument approach, as recommended by the Cochrane Handbook,^10^ since scales and check lists are not a reliable method for assessing the validity of a primary study.^11^ Randomization methods, allocation concealment, single or double blinding, intention-to-treat analysis and sample size calculations were also recorded.

### Data analysis

Analysis was undertaken according to Cochrane Collaboration guidelines. Metaanalysis for each outcome was performed by appropriately using the Review Manager software, version 4.2 (Cochrane Collaboration, Oxford, UK). For dichotomous data, relative risk (RR) was used as the effect measurement. For continuous data, the weighted mean difference was used, in which the effect estimates from individual studies were weighted by dispersion measurements.

Heterogeneity measurement was necessary in this analysis. Inconsistency among the pooled estimates was quantified using the I2= [(Q - df)/Q] x 100% test, where Q is the chi-squared statistic and df is degrees of freedom. This illustrates the percentage of the variability in effect estimates that results from heterogeneity rather than sampling error.^10,12^

In this review, subgroup analysis was performed considering the two different ximelagatran dosage schemes.

## RESULTS

### Literature search results

All the trials were identifiable by electronic databases with no exceptions. Three randomized controlled trials satisfying the inclusion criteria were identified.^13-15^ No articles were excluded. All studies gave details concerning the method used to detect VTE as well as definitions of clinical and laboratory outcomes, which were comparable between trials.

### Characteristics of included studies

All studies were double-blinded randomized controlled trials, and it was reported in two of the studies that the randomization was performed using a computer-generated randomization list.^13,14^ No details of allocation concealment were given in any of the studies. In one study,^14^ the trial was also double-dummy. In all studies, safety analysis was performed on an "intention-to-treat" basis. Independent and blinded assessment of outcomes by a Central Adjudication Committee was reported in all studies.

The three studies involved 5,284 randomized patients, including 4,914 older persons (mean ages ranged from 66.9 to 68.0 years old). They were conducted in 115^13^ to 116^14^ centers in the United States, Canada, Israel, Mexico and Brazil^13,14^ and in 74 hospitals (or centers) in the United States and Canada.^15^ The orthopedic surgery performed was knee replacement in two studies^13,14^ and knee arthroplasty in one study.^15^

Drug administration was oral in all groups. Warfarin (target INR [International Normalized Ratio] 2.5) was started in the evening of the day of the surgery and ximelagatran in the morning after the surgery in all three studies.^13-15^ In one study,^15^ the authors used 24-mg b.i.d. oral ximelagatran. Francis et al.^13^ compared two dosages of ximelagatran (24 mg b.i.d. and 36 mg b.i.d.) and Colwell et al.^14^ used 36-mg b.i.d. ximelagatran, none of which was preceded by subcutaneous melagatran. Therefore, two subgroups were considered for comparison: "Low dose" (24 mg b.i.d.) and "High dose" (36 mg b.i.d.). The use of graduated elastic stockings was not specified in any of the three studies; planned pneumatic compression prophylaxis was an exclusion criterion in two studies.^13,14^

Safety and mortality were adjudicated by an independent committee in all studies. Deaths due to all causes and with any dosages during and after treatment were considered for analysis.

The heterogeneity tests were not significant in any of the analysis.

### Venous thromboembolism

**Figure 1:**
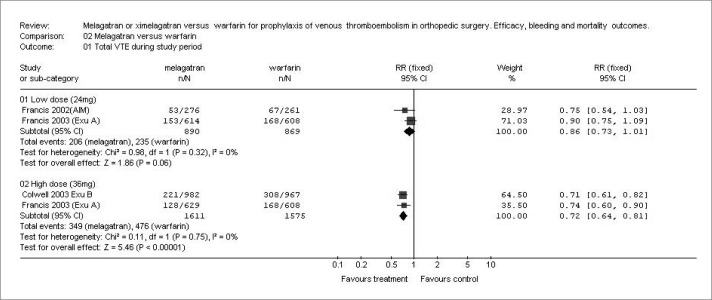
Incidence of total venous thromboembolism (VTE) with two dosages of ximelagatran versus warfarin in different studies.^13-15^

Ximelagatran showed significantly lower frequency of total VTE than did warfarin, (Figure 1), but only with the higher dosage scheme (RR: 0.72; 95% confidence interval, CI: 0.640.81; p < 0.00001). For the 24-mg subgroup, the total VTE frequency analyses were similar (RR: 0.86; 95% CI: 0.73-1.01; p = 0.06). For major VTE (RR: 0.62; 95% CI: 0.37-1.02; p = 0.06; and RR: 0.83; 95% CI: 0.58-1.18; p = 0.29) and pulmonary embolism (RR: 0.75; 95% CI: 0.19-3.00; p = 0.69; and RR: 0.81; 95% CI: 0.23-2.83; p = 0.75), there were no significant differences between the lower and higher dosages, respectively.

### Bleeding

**Figure 2:**
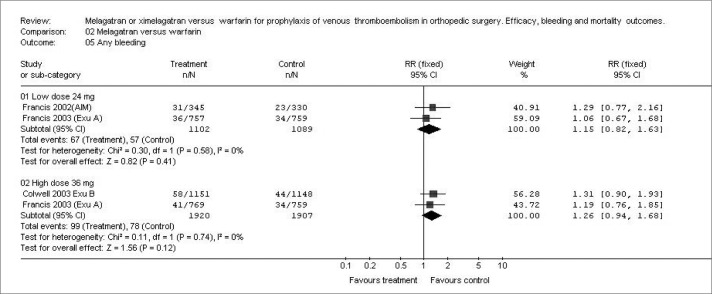
Incidence of any bleeding with two dosages of ximelagatran versus warfarin in different studies.^13-15^

**Figure 3:**
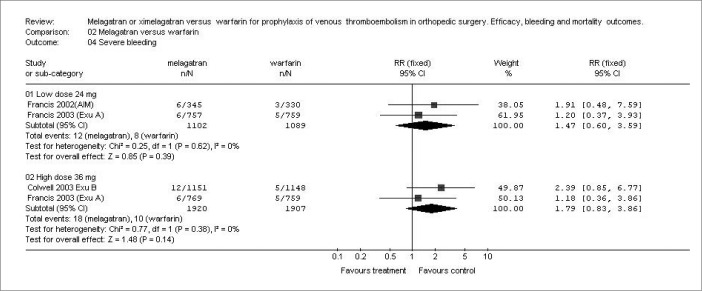
Incidence of severe bleeding with two dosages of ximelagatran versus warfarin in different studies.^13-15^

The results showed no significant differences in any bleeding (Figure 2) with either dosage (24 mg b.i.d. or 36 mg b.i.d.), respectively (RR: 1.15; 95% CI: 0.82-1.63: p = 0.41; and RR: 1.26; 95% CI: 0.94-1.68; p = 0.12), or in severe bleeding (RR: 1.47; 95% CI: 0.60-3.59; p = 0.39; and RR: 1.79; 95% CI: 0.83-3.86; p = 0.14), comparing melagatran *versus* warfarin (Figure 3). None of the studies reported the following volume measurements as mean ± SD: blood loss, volume transfused and wound drainage. Transfusion requirements were not documented in the three studies, either.

### Death

In the ximelagatran groups and warfarin groups, there were respectively eleven and eight deaths during the treatment period and eight and one deaths during the follow-up period. The causes of death were not specified, and only the frequency of fatal PE was highlighted: only one death in the ximelagatran group^14^ and one death in the warfarin group^13^ were reported. No significant difference between the groups was found regarding the frequency of deaths during the study follow-up periods with either dosage of ximelagatran: 24 mg b.i.d. (RR: 0.98; 95% CI: 0.14-6.94; p = 0.98) or 36 mg b.i.d. (RR: 1.66; 95% CI: 0.40-6.92; p = 0.49).

### Liver enzyme elevation

**Figure 4:**
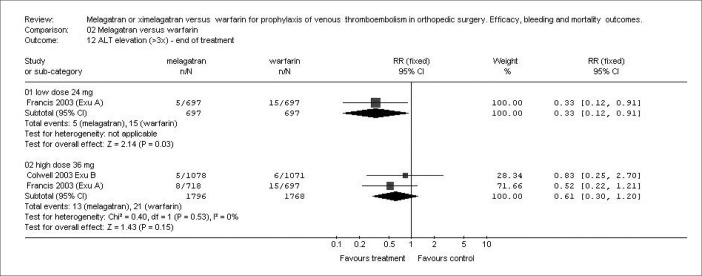
Incidence of alanine aminotransferase (ALT) elevation greater than three times the upper limit of the normal range, during the follow-up period, with two dosages of ximelagatran versus warfarin in different studies.^13,14^

The results regarding ALT elevation to three times above the upper limit of the normal range at end of treatment were similar for warfarin and the 36 mg b.i.d. ximelagatran subgroup (RR: 0.61; 95% CI: 0.30-1.20: p = 0.15). For the 24 mg b.i.d ximelagatran subgroup, the results favored ximelagatran (RR: 0.33; 95% CI: 0.12-0.91; p = 0.03), although there was only one study supporting this result. However, during the follow-up period, the 36-mg b.i.d. ximelagatran subgroup showed significantly higher frequency of persistent elevation of liver enzymes (RR: 6.97; 95% CI: 1.26-38.50; p = 0.03) (Figure 4).

## DISCUSSION

For nearly 50 years, anticoagulation therapy has been dominated by unfractionated heparin and oral vitamin K antagonists (VKA), and only more recently have low molecular weight heparins (LMWH) been introduced. The wide clinical experience accumulated with these drugs over the years has helped to develop evidence-based guidelines and define their place in the primary and secondary prevention of venous and arterial thromboembolic diseases. However, some limitations of VKA, such as slow onset of action, interaction with numerous foods and drugs, and the need for careful monitoring, plus the limitations of LMWHs, i.e. parenteral administration only, bleeding risks (particularly when administered soon after orthopedic surgery) and thrombocytopenia risk,^16^ have stimulated the search for new anticoagulants. The ideal profile of an anticoagulant should be: oral and parenteral administration; no requirements for coagulation monitoring; wide therapeutic window; appropriate elimination half-life; rapid onset of action; minimal interaction with food and other drugs; low non-specific plasma protein binding; ability to inhibit free and clot-bound thrombin; not crossing placental barrier; non-animal; easily obtainable; low cost; and antidote available. A number of new anticoagulant agents have been studied in an attempt to match this profile, including direct thrombin inhibitors, inhibitors of factor Xa, factor IXa, factor VIIa-tissue factor complex, and factor Va-factor VIIa complex.^17^ Among the new drugs is ximelagatran, the first oral thrombin inhibitor (DTI). Ximelagatran is rapidly and completely converted to its active form melagatran, which exerts antithrombotic effects. A number of characteristics have been identified in studies, such as: predictable pharmacokinetic profile that is not affected by age, ethnicity or body weight; non-interaction with food; low potential for drug interaction; and wide therapeutic window. These suggest that ximelagatran can be used with fixed-dose regimens and without coagulation monitoring, which would facilitate self-administration and adherence to treatment, particularly during the six-day postoperative period after discharge.^9^ One limitation would be the lack of an antidote in the event of severe bleeding.

Patients undergoing orthopedic surgery are at high risk of developing thromboembolic complications. The frequency of DVT in the absence of prophylaxis has been found to be 5-77% in hip replacement and 22-80% in knee replacement.^1^

In a meta-analysis on thromboembolic prophylaxis in total knee arthroplasty,^18^ comparison of several agents used in DVT prophylaxis (LMWH, warfarin, aspirin, heparin, mechanical methods, antithrombin III and dextran) with placebo, showed that for total DVT, all agents except dextran and aspirin, provided significantly better protection than did placebo (p < 0.0001). In particular, LMWH showed absolute frequencies of distal and proximal DVT of, respectively, 24.4% (95% CI: 20.3-29.0) and 5.9% (95% CI: 4.0-8.5). The American College of Chest Physicians (ACCP) recommends routine thromboprophylaxis using LMWH (at the usual high-risk dose), fondaparinux, or adjusted-dose VKA (target INR, 2.5; INR range, 2.0 to 3.0).^2^ Thus, LMWH and warfarin are good comparators for studies on the efficacy of new agents for knee surgery thromboprophylaxis.

One previous meta-analysis comparing timing of administration and treatment using melagatran/ximelagatran and LMWH in major orthopedic surgery has been published.^8^ In that study, the relationships between the efficacy, safety and timing of initial administration of the drugs were analyzed. Six studies^19-24^ with a total of 8,450 patients were included in the meta-analysis. Pooled analysis showed that melagatran/ximelagatran administered preoperatively seemed to be more effective than when administered postoperatively, regarding the effect on total DVT (respectively, RR: 0.68; 95% CI: 0.51-0.89; and RR: 1.14; 95% CI: 0.77-1.69). Conversely, preoperative administration seemed to increase the need for blood transfusions (respectively, RR: 1.08; 95% CI: 1.07-1.14; and RR: 0.95; 95% CI: 0.90-0.99) and the proportion of severe bleeding (respectively, RR: 2.51; 95% CI: 1.60-3.92; and RR: 0.85; 95% CI: 0.52-1.40). The authors concluded that there was a relationship between venous thromboembolic or hemorrhagic risk and the timing of the first administration of melagatran/ximelagatran and suggested that, in terms of benefit-risk ratio, there should be postoperative administration of melagatran/ ximelagatran for thromboprophylaxis in major orthopedic surgery.

In another meta-analysis^9^ including the same six articles but now involving 10,051 patients, similar findings were obtained. In comparison with postoperative ximelagatran, LMWH had a significantly lower rate of VTE (odds ratio, OR: 0.68; 95% CI: 0.56-0.82; p < 0.001), with no difference in bleeding rate (OR: 1.09; 95% CI; 0.62-1.94; p = 0.76) in hip surgery or knee surgery. Compared with ximelagatran administered immediately before surgery, LMWH had a significantly higher rate of VTE in both hip surgery (OR: 1.87; 95% CI: 1.20- 2.92; p = 0.006) and knee surgery (OR: 1.49; 95% CI: 1.14- 1.93; p = 0.003), but less bleeding (respectively, OR: 0.30; 95% CI: 0.17-0.53; p < 0.001; and OR: 0.71; 95% CI: 0.30-1.67; p = 0.43). Thus, it was demonstrated that benefits in VTE prevention using ximelagatran were gained at the expense of an increased risk of serious bleeding. The studies included presented significant heterogeneity, and had to be grouped according to surgical subtype and also according to the timing of melagatran/ximelagatran administration, in order to reach meaningful conclusions.^8,9^

In the present review, the comparison with warfarin had less heterogeneity because the timings of administration of the study drug and the comparator were similar, ximelagatran was always administered without previous subcutaneous melagatran, the primary outcome (DVT) was diagnosed by bilateral venography in most studies, and the type of surgery was always knee surgery. Heterogeneity was present only in relation to the ximelagatran dosages, which warranted subgroup analysis. Although the number of patients studied was around half of the number in the comparison with LMWH, the total population included was significant (total = 4,914). Thus, in the present systematic review, ximelagatran had a significantly lower rate of total VTE than did warfarin in knee surgery, but only when it was used in higher dosages (36 mg b.i.d.). No difference was found in relation to major VTE or PE. This efficacy was not gained at the expense of increased bleeding, which was similar to warfarin at any ximelagatran dosage scheme. Perhaps the starting time for ximelagatran (in the morning after surgery) was beneficial for preventing bleeding complications. Safety analysis was impaired because of the lack of information on mean and standard deviations of blood loss volumes, blood drainage, blood volume transfused, and bleeding index. Contacts with authors through the AstraZeneca Medical Department in Brazil were unsuccessful in obtaining these data.

The frequency of ALT of more than three times the upper limit of the normal range at the end of the study period was practically the same for both treatments. However, the frequency of ALT in the "high-dosage" ximelagatran subgroup was greater than in the warfarin group during the follow-up period (four to six weeks after surgery).^13,14^ Liver enzyme elevation has been of particular concern in long-term treatment trials using ximelagatran (6% in Sportif V,^25^ 6.3% in Sportif III^26^ and 6% in Thrive III^27^), which characteristically increased after 2-6 months. Thereafter, the liver enzyme levels returned to the baseline without clinical sequelae, regardless of whether or not the drug was continued, with a median to normalization of 129 days reported from Thrive III.^28^ In a post-hoc analysis,^29^ the following risk factors for ALT elevation were identified: treatment of acute coronary syndrome, treatment for acute VTE, female, low body mass index (< 27), and concomitant use of statins. Asian patients were at decreased risk. In the long-term trials, 0.37% of patients receiving ximelagatran and 0.08% of patients taking comparators presented associated bilirubin levels of twice the normal value within the next month, which raised some concerns about evolution to liver failure for these patients^29^ (*see authors’ note below*). Therefore, for long-term treatment, it would be important to have an algorithm for preventing ALT elevation and management for better results. The cause of this ALT elevation cause has not yet been clarified in the literature. It has been stated that ALT elevation could be due to anything from cell toxicity or nuclear effect with protein induction to changes in membrane permeability.^28^ ALT elevation occurs not only with LMWH but also with warfarin, as demonstrated in the two trials included in the present review^13,14^

Cardiovascular events were not reported in any of the three trials included in the present review. According to Gulseth,^29^ disease-related adverse effects on coronary arteries occurred more frequently in the ximelagatran group: the frequency of myocardial infarction was 0.6% in the ximelagatran group and 0.21% in the warfarin group (p = 0.04951),^29^ and myocardial infarction, angina and ischemia were also more frequent in the ximelagatran group (0.75%) than in the warfarin group (0.26%). In our meta-analysis, the mortality rates were similar in the comparison with warfarin.^13,14^ Except for PE, most deaths were not attributed to the treatment itself, as judged by the trials Central Committee. Because there are few reports and low incidence, further studies are necessary for definite conclusions to be reached.

The main competing agent for pharmacological prophylaxis in major orthopedic surgery is fondaparinux, which has recently received approval from the Food and Drug Administration (FDA) in the United States, for use in such cases.^2^ In a meta-analysis, fondaparinux *versus* enoxaparin for preventing VTE in major orthopedic surgery significantly reduced the incidence of VTE by day 11 (odds reduction: −55.2; 95% CI: −63.1, −45.8; p < 0.001), which was consistent for all types of surgery and subgroups. However, severe bleeding was more frequent in the fondaparinux-treated group (p = 0.008), although the rates of any bleeding or clinically relevant bleeding were similar.^30^

Nevertheless, the risk of VTE has been estimated in all these studies basically during short-duration prophylaxis programs. It is known that the risk of symptomatic non-fatal VTE occurring within three months after knee or hip replacement in patients who received short-duration (7-10 days) anticoagulant prophylaxis affects approximately one in every 32 patients, and that fatal pulmonary embolism occurs in one in every 1000 patients, and is more frequent in hip surgery.^31^

## REVIEWERS’ CONCLUSIONS

### Implications for practice

In the light of the currently available information on ximelagatran/melagatran *versus* warfarin for preventing VTE, ximelagatran appears to be more effective and safe when used in higher dosages (36 mg b.i.d.) for total VTE. Any occurrences of bleeding, severe bleeding and death were similar to findings with warfarin, with any ximelagatran dosage scheme. However, ALT remained significantly more frequently elevated in patients receiving the 36-mg b.i.d. dosage scheme during the follow-up period.
